# The Structural Evolution and Mechanical Properties of Semi-Aromatic Polyamide 12T after Stretching

**DOI:** 10.3390/polym14224805

**Published:** 2022-11-08

**Authors:** Yuting Shang, Hongchuan Lou, Wei Zhao, Yuancheng Zhang, Zhe Cui, Peng Fu, Xinchang Pang, Xiaomeng Zhang, Minying Liu

**Affiliations:** 1 School of Materials Science and Engineering, Henan Key Laboratory of Advanced Nylon Materials and Application, Engineering Laboratory of High Performance Nylon Engineering Plastics of China Petroleum and Chemical Industry, Zhengzhou University, Zhengzhou 450000, China; 2 The State Key Laboratory of Polymer Materials Engineering, Polymer Research Institute of Sichuan University, Chengdu 610065, China; 3 Jinguan Electric Co., Ltd., Nanyang 473000, China

**Keywords:** polyamide, structural evolution, mechanical properties

## Abstract

The development of semi-aromatic polyamides with excellent mechanical properties has always been a popular research avenue. In this work, the semi-aromatic polyamide 12T (PA12T) with the maximum tensile strength of 465.5 MPa was prepared after stretching at 210 °C 4.6 times. Wide-angle X-ray diffraction (WAXD) and small-angle X-ray scattering (SAXS) were used to characterize the structural evolution of semi-aromatic polyamide 12T (PA12T) after stretching at different stretching temperatures and stretching ratios. The formation mechanism of this change in mechanical properties was investigated from different aspects of the aggregated structure such as crystal morphology, crystal orientation and crystallinity. The relevant characterization results show that the crystal structure, crystal orientation and crystallinity of PA12T were the highest when the sample was pre-stretched at 210 °C, which is crucial for improving the mechanical properties of PA12T. These findings will provide important guidance for the preparation of polymer materials with excellent mechanical properties.

## 1. Introduction

Semi-aromatic polyamides are important engineering plastics with excellent comprehensive properties, as shown in Table S1 in the supporting information [1,2,3,4,5,6,7]. Polydodecanediamine terephthalamide (PA12T) is a new kind of semi-aromatic polyamide polymerized from 1,12-dodecanediamine (DA12) and terephthalic acid (PTA) [1,8]. Compared with other semi-aromatic polyamides, such as commercialized PA6T and PA9T, PA12T possesses much better melting property and lower water adsorption, due to more methylene groups in the backbone [9,10,11,12]. Furthermore, DA12, the monomer of PA12T, is mainly derived from the by-product of petroleum through the method of microbial fermentation, which makes PA12T environmentally friendly [13]. Therefore, PA12T has great potential for application as high-performance materials in various fields, such as aerospace, automobiles and electronics [14,15]. 

Compared with other materials, the mechanical properties of polymers are always undesirable for usage as collision protection or structure materials. To date, several approaches have been proposed for improving mechanical properties of polymers, such as blending with reinforcing fillers and regulating intrinsic crystal structures [16,17,18,19,20,21]. Among them, regulating intrinsic crystal structures of polymers is a great method for improving mechanical properties due to the low cost and regardless of interfacial compatibility between polymer and filler [16,22,23,24].

Stretching is an effective way to simultaneously improve the crystallinity and molecular chain orientation of polymers, which play critical roles on enhancing mechanical properties [25,26,27,28,29,30,31,32]. Cai et al. used WAXD and SAXS techniques to study the structural evolution of polyamide 1212 (PA1212) during stretching at different temperatures [33]. The results show that PA1212 has the maximum tensile strength after pre-stretching at 140 °C, due to the highest crystal orientation and crystallinity. Cui et al. used WAXD and DSC to study the crystal structure of PA510 films after uniaxial stretching at different stretching ratios [34]. The results show that the crystallinity and orientation of molecular chain significantly increased with increasing the stretching ratio, due to the strain-induced recrystallization during the stretching process. As expected, mechanical properties of PA510 were enhanced remarkably.

Although the crystal transition behavior and its relationship with mechanical properties of conventional polyamides have been extensively studied in recent years, the correlational research on semi-aromatic polyamides is still insufficient. In this work, by using PA12T as the research object, the effects of stretching temperature and stretching ratio on its crystal structure and crystal transition behavior are systematically investigated, and the results will provide important guidance for fabricating high-performance semi-aromatic polyamides and expanding their applications.

## 2. Experimental Section

### 2.1. Materials and Preparation Process

PA12T (Tg = 140 °C, Tm = 315 °C) was provided by the Junheng Biotechnology Co., Ltd., Henan, China. PA12T was dried in a vacuum oven at 120 °C for 12 h before use. PA12T films with thickness of 0.2 mm were prepared by compression molding at 340 °C and 10 MPa for 5 min. Then, the melting films were dropped into ice water for rapid cooling. Lastly, standard dumbbell specimens with width of 4 mm, thickness of 0.2 mm and length of 25 mm were cut from the films for stretching experiments. 

The stretching was carried out in an electromechanical universal testing machine equipped with a heating box. Specimens were stretched under the constant rate of 10 mm/min with stretching ratios of 1.0, 1.6, 2.2, 2.8, 3.4, 4.0 and 4.6 and stretching temperatures of 90, 150, 180, 210, and 240 °C. After stretching, specimens were cooled down to room temperature for subsequent tests.

### 2.2. Characterization

The mechanical properties of uniaxially stretched films were measured at room temperature using the electromechanical universal testing machine (MTS Industrial Systems Co, Shenzhen, China) with tensile rate of 10 mm/min along the stretching direction. Thermal analysis of specimens was conducted using differential scanning calorimetry (DSC 214, NETZSCH) under nitrogen atmosphere with heating rate of 10 °C/min. The melting point (Tm) and melting enthalpy (ΔHm) were determined. Wide-angle X-ray diffraction (XRD) was performed on Smart Lab SE (Riku Corporation, Tokyo, Japan), and the scanning speed was 3 °/min. The interplanar spacings (d) were calculated using Bragg’s equation:

2dsinθ = nλ,(1)

where λ (=0.154 nm) is the wavelength of the Cu Kα X-ray radiation.

Two-dimensional wide-angle X-ray scattering (2D-WAXS) and two-dimensional small-angle X-ray scattering (2D-SAXS) were all performed on the Xeuss 2.0 system (Xenocs SA, Sassenage, France) with Cu Kα radiation. The wavelength of X-ray radiation was 0.154 nm, and the detector was perpendicular to the surfaces of specimens. The detector-to-sample distances were 116 mm and 2480 mm, and the exposure time was 20 min. Dynamic mechanical analysis (DMA) was performed on a TA Q800 instrument (TA Instruments Co, New Castle, DE, USA) under N2 atmosphere at a frequency of 2 Hz with a heating rate of 3 °C/min from −100 °C to 200 °C under the tensile mode.

## 3. Results and Discussion

### 3.1. Effect of Uniaxial Stretching on Mechanical Properties

**Figure 1:**
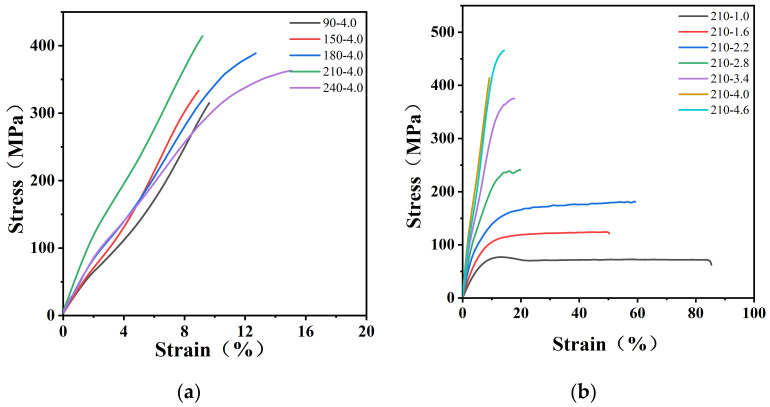
Stress–strain curves of PA12T samples with (**a**) different stretching temperatures; (**b**) different stretching ratios.

**Figure 2:**
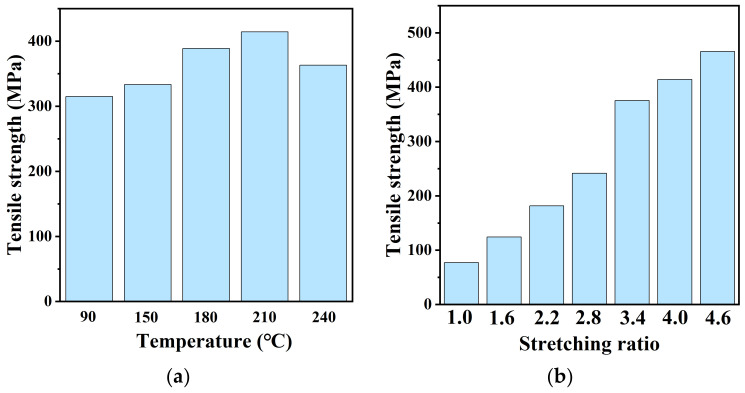
Tensile strengths of samples with (**a**) different stretching temperatures; (**b**) different stretching ratios.

PA12T films were uniaxially stretched at different ratios and temperatures. The stretching temperatures were 90, 150, 180, 210 and 240 °C, and the stretching ratios were 1.0, 1.6, 2.2, 2.8, 3.4, 4.0 and 4.6. After stretching, the specimens were cooled down to room temperature. The mechanical properties of the uniaxially stretched PA12T films were measured at room temperature, and the stress–strain curves are shown in Figure 1a,b. It can be seen that the tensile strengths of the stretched PA12T films were much higher than that of the original samples (about 77.1 MPa). Additionally, the samples prepared at higher temperatures at the same stretching ratio had greater tensile strength. For example, the samples stretched at 210 °C had the highest mechanical strength. As shown in Figure 2a,b, at the constant stretching temperature of 210 °C, the tensile strength of the samples increased with increasing the stretching ratio, and the maximum tensile strength of 465.5 MPa was obtained at a ratio of 4.6, which was 503% higher than that of the unstretched sample.

In the process of high-performance fiber and high-performance film production, parameters such as stretching temperature and stretching ratio are very important for the improvement of mechanical properties [19,20,25,35,36,37,38,39,40,41,42,43]. Therefore, it is very important to study the evolution of the aggregated state structure (such as crystal structure, crystal orientation) of PA12T under different stretching conditions and reveal the enhancement mechanism of its mechanical property.

### 3.2. Effect of Uniaxial Stretching on Crystal Structure

**Figure 3:**
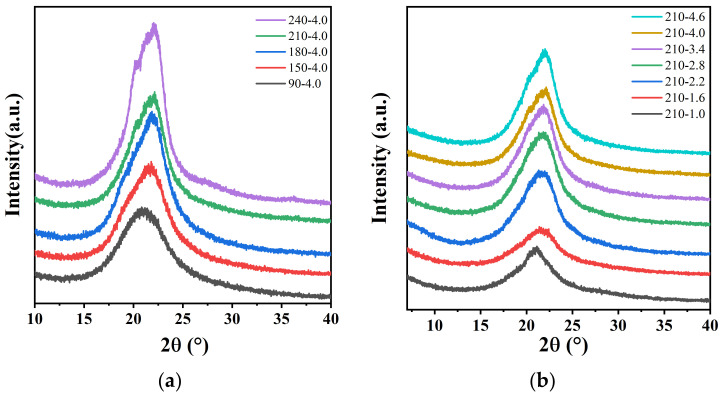
WAXD patterns of PA12T samples stretched at (**a**) different stretching temperatures; (**b**) different stretching ratios.

First, the effects of stretching temperature and stretching ratio on the crystal structure of PA12T after uniaxial stretching were investigated with XRD, as shown in Figure 3. We can clearly see that the unstretched PA12T film sample has only one characteristic diffraction peak at the position of 2θ = 21.04°, which is the characteristic diffraction signal of the (100) crystal plane of γ-crystal [44,45]. From Figure 3a, it can be seen that the diffraction peak shifts to the right with increasing stretching temperature, which means the d-spacing decreases, and its minimum appears at 210 °C. The positions of the diffraction peaks and the corresponding d-spacings are summarized in Table 1. The reason for this phenomenon is that the increases in stretching temperature and ratio facilitate the movement and alignment of molecular chains along the stretching direction and finally form more closely arranged and ordered crystals.

**Figure 4:**
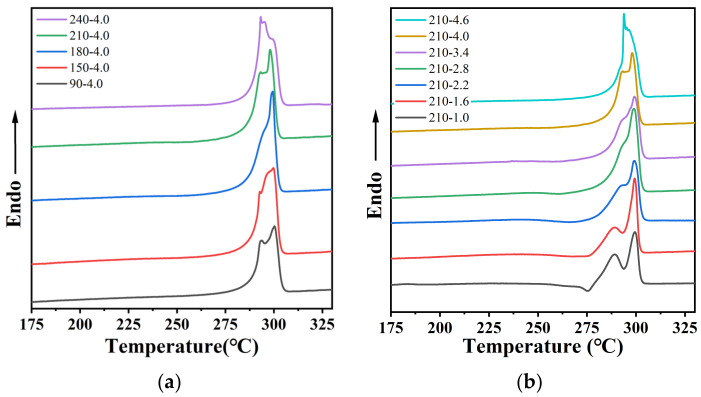
DSC melting curves of PA12T samples with (**a**) different stretching temperatures at stretching ratio of 4; (**b**) different stretching ratios at stretching temperature of 210 °C.

The crystallinity of PA12T samples were investigated with DSC, and the results are shown in Figure 4 and Table 1. It can be seen that the stretched PA12T samples possess multiple melting peaks and that melting points slightly reduce with increasing stretching temperature or ratio. The appearance of multiple melting peaks and the decrease in melting points are mainly attributed to the instable crystal morphology caused by crystal rupture during the stretching process [46,47,48,49]. With the increase of stretching temperature and stretching ratio, this phenomenon becomes much more significant due to the improved motility of segments, and therefore, crystal rupture is more likely to occur. Similarly, at the same temperature, the greater the stretching ratio, the greater the deformation along the stretching direction, and the more crystal rupture will also occur. Furthermore, based on the results shown in Table 1, the melting enthalpies of the PA12T samples gradually increase with increasing stretching temperature and stretching ratio, due to the stretching-induced recrystallization process. However, when the stretching temperature is 240 °C, the melting enthalpy is decreased because of the increased crystal rupture and the restricted recrystallization process at high temperature.

**Figure 5:**
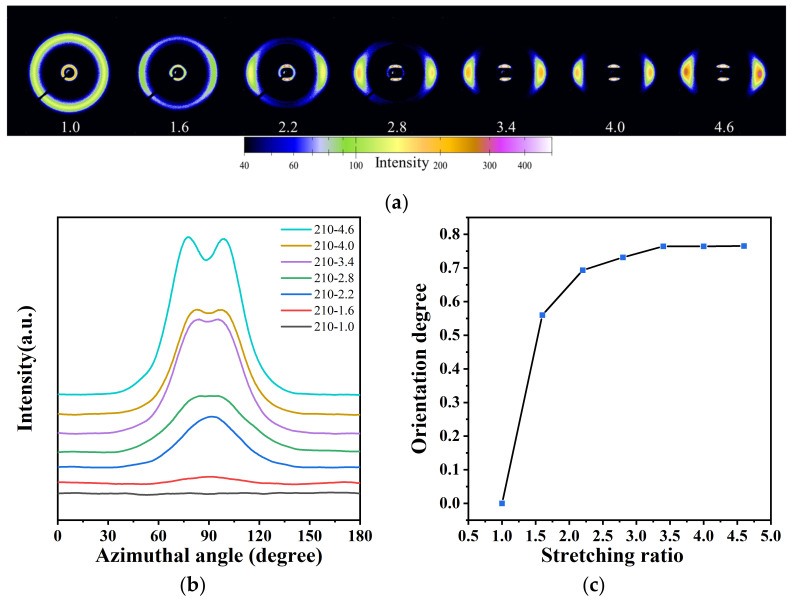
(**a**) 2D-WAXD diffraction patterns of PA12T samples with different stretching ratios; (**b**) azimuthal distribution of PA12T samples with different stretching ratios; (**c**) relationship between crystal orientation and stretching ratio.

Next, the effects of stretching temperature and stretching ratio on the orientation of crystals were investigated by 2D-WAXD. As shown in Figure 5a, before stretching, the 2D-WAXD pattern of the original PA12T sample shows a distinct Debye–Scherrer ring, which is a typical isotropic crystal diffraction [50,51]. The Debye ring is assigned to the (100) lattice plane of γ-crystal. With increasing the stretching ratio, the diffraction pattern gradually changes from ring to shorter diffraction arcs located on the equator. This phenomenon is mainly attributed to the orientation adjustment of crystals under the tensile stress, including the distortion, rotation and sliding of the lamellae [45]. In particular, at the stretching ratio of 4.6, it can be observed that the diffraction arcs change to a clear diffraction spot on the equator, which indicates a highly oriented state of the crystals within PA12T along the stretching direction, as shown in Figure 5b,c. The diffraction intensity also increases significantly due to the stretching-induced recrystallization of PA12T, which is consistent with the results of DSC shown in Table 1.

**Figure 6:**
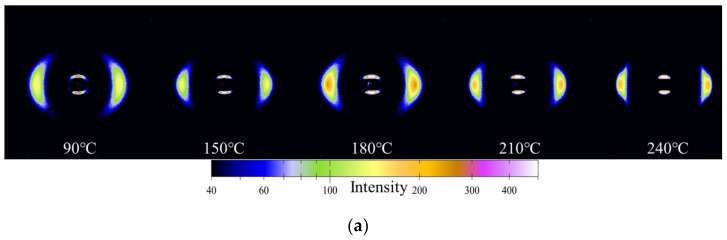

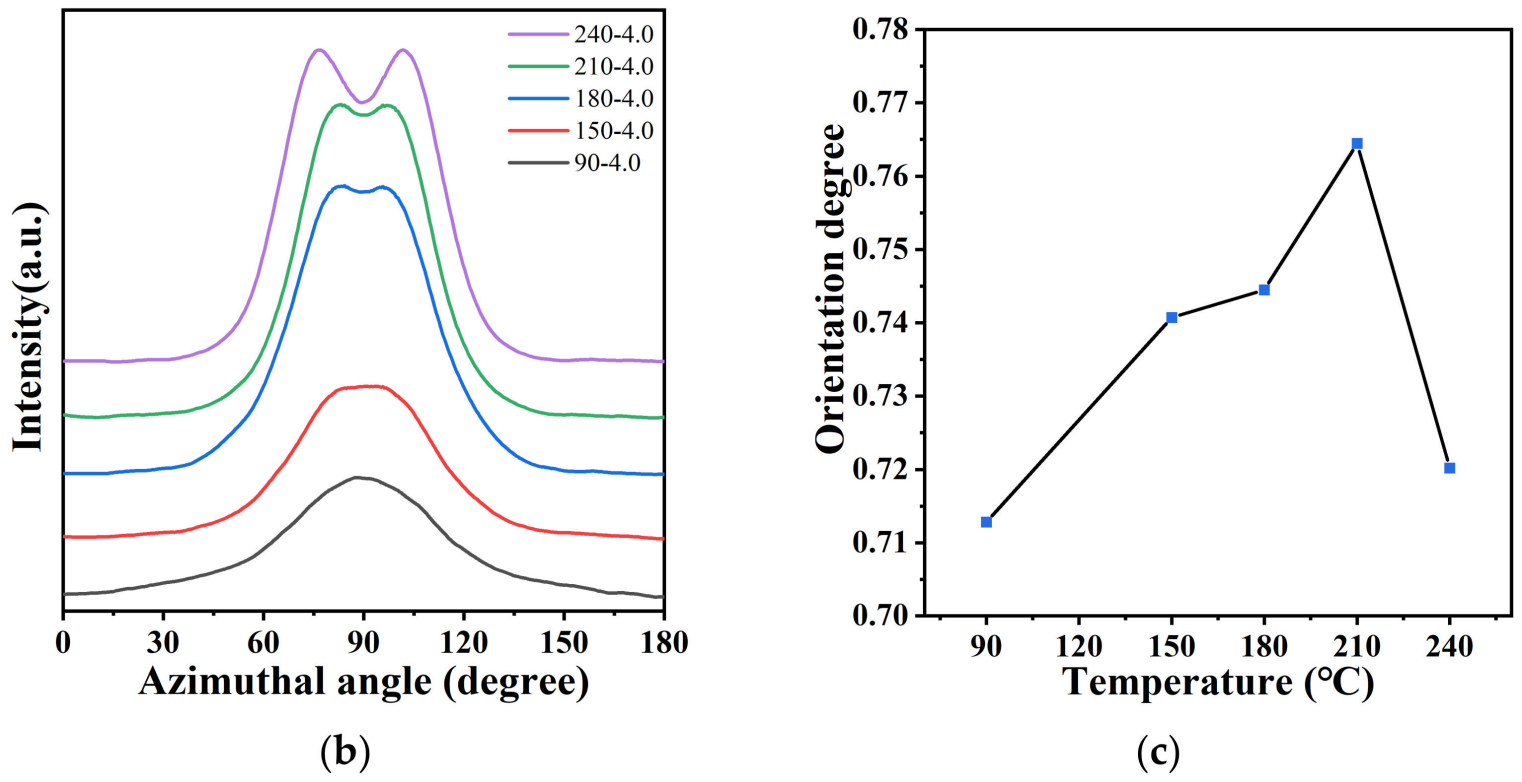
(**a**) 2D-WAXD diffraction patterns of PA12T films at different stretching temperatures; (**b**) azimuthal distribution of PA12T films at different stretching temperatures; (**c**) relationship between crystal orientation degree and stretching temperature.

As shown in Figure 6, when the stretching ratio is 4, the crystal orientation of PA12T increases as the stretching temperature increases from 90 °C to 210 °C, but when the temperature reaches 240 °C, the degree of crystal orientation decreases significantly. The reason for this phenomenon is as follows: Below 210 °C, the mobility of the molecular chains gradually improves with increasing temperature, while the relaxation behavior can still be restricted, and as a result, crystal orientation improves with increasing temperature. When the temperature increases to 240 °C, relaxation behavior of the molecular chains becomes more significant and inhibits the orientation of the crystal along the stretching direction, which leads to a decrease in crystal orientation. Therefore, crystal orientation of PA12T is determined by both the mobility and relaxation effects of molecular chains during the stretching process [33,44,52,53].

**Figure 7:**
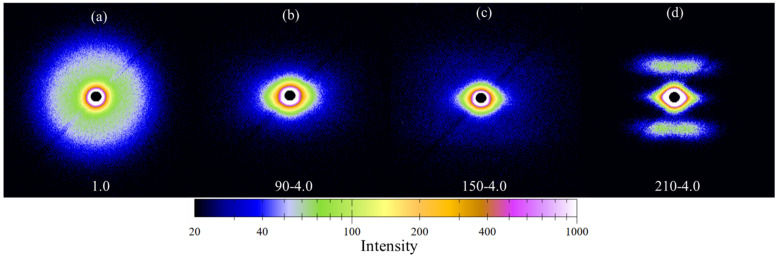
2D-SAXS patterns of PA12T samples (**a**) unstretched, (**b**–**d**) stretching ratio is 4.0 and stretching temperatures are 90 °C, 150 °C and 210 °C.

Next, 2D-SAXS was used to further investigate the crystal evolution during stretching. PA12T samples with different stretching temperatures and ratios were prepared for characterization. As shown in Figure 7a, the SAXS pattern of the original PA12T sample is a broad isotropic ring, indicating that the lamellae are randomly distributed in crystal [54]. As shown in Figure 7b, after stretching at 90 °C at stretching ratio 4, an anisotropic and multipoint pattern can be observed that is caused by the slippage of lamellae during the stretching process, but the orientation structure is not sufficient. As shown in Figure 7c, with further increasing the temperature to 150 °C, this phenomenon becomes much more significant due to the improved mobility of lamellae as well as molecular chains, but the lamellae still cannot be oriented entirely. As shown in Figure 7d, under the same stretching ratio, when the stretching temperature is 210 °C, two new scattering peaks along the stretching direction can be observed, and no multipoint patterns appear, indicating that at 210 °C, the new lamellae are generated from the crystallization process, and the lamellae are already parallel to the orientation direction [48,54,55,56,57,58].

**Figure 8:**
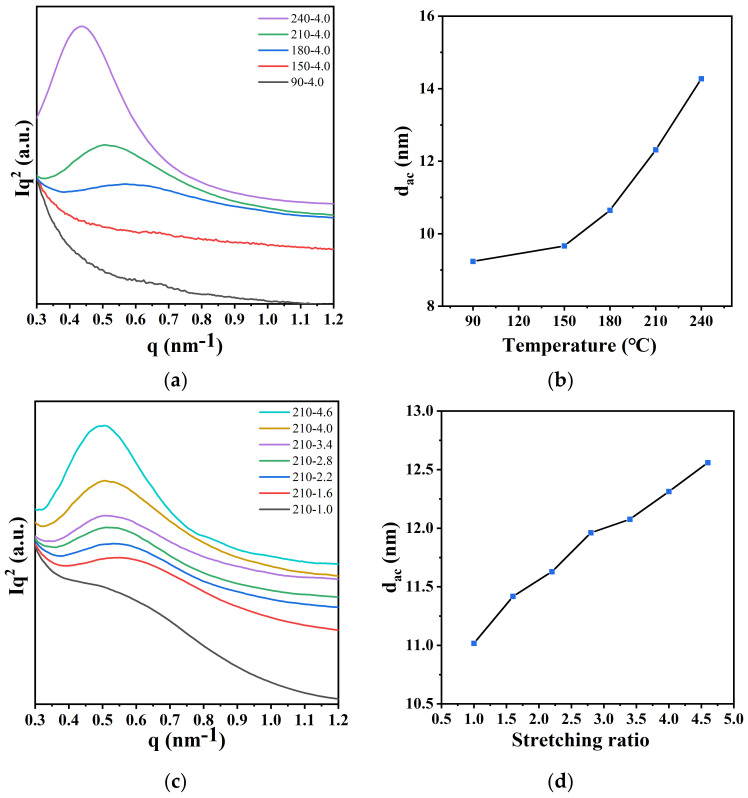
(**a**) 1D-SAXS intensity distribution curves of PA12T films with different stretching temperatures; (**b**) relationship between the d_ac_ and stretching temperature; (**c**) 1D-SAXS intensity distribution curves of PA12T samples with different stretching ratios; (**d**) relationship between the d_ac_ and the stretching ratio.

In order to investigate the evolution of the lamellar long spacing of PA12T samples after stretching at different temperatures and ratios, the 1D-SAXS intensity distributions of the samples along the stretching direction are shown in Figure 8a,c. The abscissa corresponding to the peak is defined as q_max_. The long spacing d_ac_ is defined as the average thickness of the lamella and the amorphous layer between two adjacent lamellae. The scattering intensity is multiplied by q^2^ for data processing (i.e., Lorentz correction) [44,45]. Lamellar long spacing d_ac_ can be calculated using the following formal formula, and results are shown in Figure 8b,d:

d_ac_ = 2Π/q_max_, (2)

As shown in Figure 8a,b, the scattering peaks shift towards lower q values, and the intensities of peaks increase with increasing stretching temperature, which indicates that the molecular arrangement is more regular [33,49]. The long spacing of PA12T samples also increases with the increase in temperature, which means that PA12T is more conducive to the formation of more ordered and thicker lamellae at high temperature.

**Figure 9:**
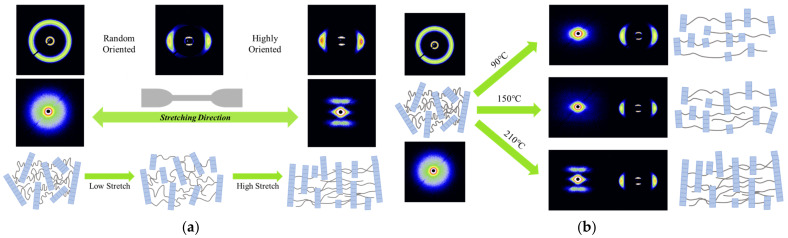
Schematic model of structure evolution during uniaxial stretching at (**a**) different stretching ratios; (**b**) different stretching temperatures.

As shown in Figure 8c,d, with increasing the stretching ratio, the long spacing of PA12T samples increases. In detail, during this process, the lamellar are tilted and slipped under the action of tensile stress, and some crystals are broken, but at the same time, with the alignment of molecular chains along the stretching direction, a new crystalline structure with more ordered and thicker structure is also formed. Therefore, the long spacing of PA12T samples increases with increasing stretching ratio. Based on the results, a schematic model of structure evolution is proposed in Figure 9a,b.

### 3.3. Effect of Uniaxial Stretching on Dynamic Thermomechanical Properties

**Figure 10:**
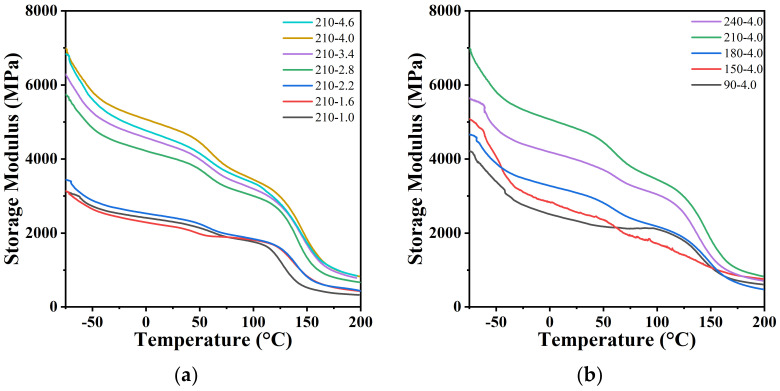

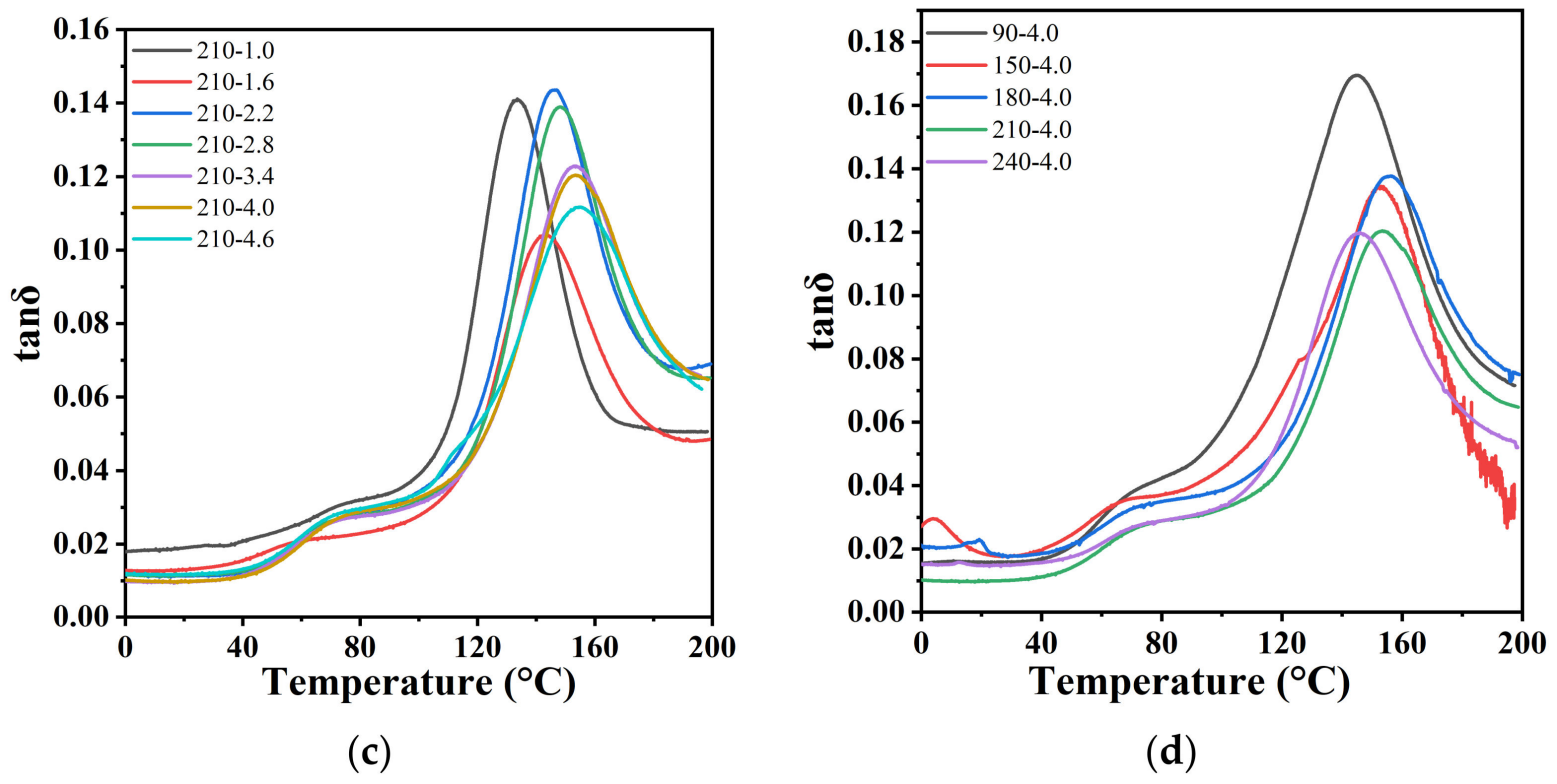
(**a**,**b**) Storage modulus–temperature curves and (**c**,**d**) tan δ–temperature relationship curves of PA12T samples with different stretching ratios or temperatures.

The relationship between stretching temperatures/stretching ratios and the storage moduli of the PA12T samples as well as the relationships between temperature/ratio and tan δ are shown in Figure 10, and the detailed values are shown in Table 2. With increasing stretching ratio and stretching temperature, the storage modulus and peak position of tan δ (α-transition temperature) increases significantly, and δ decreases. The reason for this phenomenon is that molecular chains are closely arranged in parallel after stretching, and as a result, the orientation degree and rigidity of the PA12T samples are increased. On the contrary, the toughness is decreased [59]. However, when the stretching temperature is 240 °C, the orientation of the molecular chains cannot remain, and the recrystallization process is also restricted, so the storage modulus and α-transition temperature of PA12T samples are decreased.

## 4. Conclusions

In this study, uniaxially stretched PA12T samples with different stretching ratios and temperatures were prepared, and the evolution of the structure and mechanical properties were investigated in detail. Below 210 °C, increases in the stretching temperature and the stretching ratio are both beneficial to the improvement of the orientation degree, crystallinity and mechanical properties of PA12T samples. When the stretching temperature is 210 °C and the stretching ratio is 4.6, the tensile strength of PA 12T is 465.5 MPa, which is 503% higher than that of the unstretched samples. When the stretching temperature is 240 °C, mechanical properties decrease due to the relaxation effect of molecular chains and restricted recrystallization process. Therefore, the relationship between the stretching process and morphology/properties of PA12T is well established, and the results can provide important theoretical and experimental guidance for the design or modification of polymer materials. 

## Figures and Tables

**Table 1:** D-spacing and melting enthalpy of uniaxially stretched PA12T films.

Stretching Temperature (°C)	Stretching Ratio	2θ(°)	d-Spacing (nm)	Melting Temperature (°C)	Melting Enthalpy (J/g)
90	4.0	21.05	0.421	300.3	58.0
150	4.0	21.63	0.410	299.8	71.9
180	4.0	21.96	0.405	299.4	71.6
210	4.0	22.07	0.402	298.0	72.1
240	4.0	22.03	0.403	295.1	76.9
210	1.0	21.01	0.423	299.5	61.8
210	1.6	21.47	0.414	299.3	63.3
210	2.2	21.6	0.412	299.2	67.8
210	2.8	21.74	0.408	299.0	69.0
210	3.4	21.76	0.408	299.1	67.5
210	4.0	22.07	0.402	298.0	72.1
210	4.6	22.09	0.402	293.8	67.4

**Table 2:** The α transition temperature of PA12T samples prepared at different stretching ratios and stretching temperatures.

Stretching Temperature (°C)	Stretching Ratio	α Transition Temperature (°C)
90	4.0	145.4
150	4.0	152.9
180	4.0	156.1
210	4.0	153.6
240	4.0	146.0
210	1.0	133.6
210	1.6	142.6
210	2.2	146.4
210	2.8	148.0
210	3.4	153.0
210	4.0	153.6
210	4.6	155.4

## Data Availability

The raw/processed data required to reproduce these findings cannot be shared at this time as the data form part of an ongoing study. However, they will be made available on request.

## Supplementary Materials

The following supporting information can be downloaded at: https://www.mdpi.com/article/10.3390/polym14224805/s1, Table S1: Mechanical properties of different polyamides [1,2,3,4,5,6,7].
