# Supplementary material for: The Structural Evolution and Mechanical Properties of Semi-Aromatic Polyamide 12T after Stretching
Source: Polymers (Basel). 2022 Nov 8;14(22):4805. doi: 10.3390/polym14224805 (PMC9693562; doi:10.3390/polym14224805)
Supplement: Supplementary file 1 [file polymers-14-04805-s001.zip › polymers-1983229-supplementary.pdf]

polymers

MDPI

Table S1. Mechanical properties of different polyamides.

Samples	Tensile strength (MPa)
PA12T	95 [1]
PA12T film	77
PA12T uniaxially oriented film	466
PA9T	92 [1]
PA6	75 [54]
PA11	46 [55]
PA12	44 [56]
PA66	72 [57]
PA1010	46 [58]
PA1212	54 [59]
